# 

**Published:** 1889-01

**Authors:** 


### TABLE OF CONTENTS.

### ORIGINAL AND SELECTED.

Typhoid Fever, by J. P. Stevens, M. D. .... 1
Surgery of the Germatous growhths of the nasal cavities, by Geo. Hope, M. D., N, Y .... 10
Report of operation for Hæmatoma, by Prof. J McFadden Gaston, M. D. .... 12
Enlarged Tonsils, what shall we do for them? 14

### ABSTRACTS, &c.

Some observations after 1,000 operations for Hæmorrhoids .... 15
Laryongology and Rhinology, under the charge of C. E. Sajous, M. D. .... 20
Diphtheria and Scarlet Fever; The antagonism of Cocaine .... 22
How to treat the Eye with a cinder in it .... 23
Hot Mustard Baths in Pneumonia .... 24
Artesian Wells in Memphis; Chloroform in Labor .... 25
The Medicines that can be given Nursing Mothers; Uses of Gelsemium Semper-virins .... 27
The Local Treatment of Diphtheria by Vlemick's Solution, &c.; The relation of Masturbation to Stricture of Urethra .... 28

Thuya Occidentalis Epithelioma of the Larynx and Pharynx; Hypnotism at Berlin; Eruptions due to Antipyrine; The Menses .... [illegible]

For Recent Prolap. Ut.: Chlo. of Am. in Neuralgia; Treatment of Erysipelas .... [illegible]

Treat. of Typhoid Fever by Carb. Ac.; Croup. [illegible]

Antifebrin; Sum. of gen. treat. of Syphilis; Hot Water in Epistaxis; Peach Leaf Poultc.

### SCIENTIFIC.

Cur. Chinese Notions; The nearest star; Electric Paostration .... 33

Lightning by means of Accumulators; A large Organ; Eels. &c. .... 34

### EDITORIALS, &c.

Our New Pub. House; The Clergy and Doctors [illegible]

The Ed. of Cin. Med. Jour. remarks; Pictures of Sir M. McKenzie and Pasteur .... [illegible]

Advertisers, &c .... 37-[illegible]

Books .... [illegible]

Special Notes .... [illegible]

